# A Closer Look at Angiitis of the central nervous system

**DOI:** 10.17712/nsj.2017.4.20170052

**Published:** 2017-10

**Authors:** Cheng Wan, Hua Su

**Affiliations:** *From the Department of Nephrology, Union Hospital, Tongji Medical College, Huazhong University of Science and Technology, Wuhan, China*

## Abstract

Angiitis of the central nervous system (ACNS) is a rather new disease entity that is defined as vascular inflammation limited to the central nervous system and was formally nominated in 1959. Etiologically, it can be divided into primary and secondary ACNS. However, the potential pathogenesis of ACNS remains unclear. The clinical presentation is variable, and there is no consensus concerning its diagnosis and management. Although the incidence is relatively low, ACNS is still a life-threatening condition. It is essential to get a comprehensive and updated understanding of the disease. In this paper, we reviewed the history, definition, classification, pathogenesis, and clinical manifestations of ACNS. In addition, we focused on the latest investigations and viewpoints regarding the diagnosis and treatment of ACNS.

Angiitis of the central nervous system refers to the vasculitis limited to the central nervous system (CNS). It is an uncommon genre and was only nominated about half a century ago by Cravioto and Feign.^1^ Actually, the earliest case report of ACNS dated back to 1922, but at that time, it was considered as an unknown form of arteritis.^2^ It was first proposed to be a separate clinicopathological entity in 1959.^1^ Most cases had fatal outcomes until the first publication of sustained clinical remission induced by a combination therapy of cyclophosphamide and glucocorticoids. Because of the insufficient insight into the pathogenesis of the disease, variable clinical manifestations and the absence of sensitive biomarkers of the disease’s activity, ACNS remains a significant challenge to clinicians.

## Classification of ACNS

Etiologically, ACNS can be categorized into primary ACNS (PACNS) and secondary ACNS (SACNS). The PACNS is angiitis confined to the CNS without any causative systemic diseases. In contrast, SACNS occurs in the setting of identical underlying diseases, such as systemic vasculitis, and autoimmune or infectious diseases.^3^,^4^

## Primary ACNS

The PACNS is a rare entity of unknown etiology that is restricted to the brain, meninges and spinal cord. It is a distinctive form of CNS single-organ vasculitis and is not an isolated manifestation from systemic vasculitis.^5^

## Adult PACNS

The subclassification of adult PACNS has not come to an agreement up to now. There were 3 subtypes of PACNS: granulomatous ACNS (GACNS), benign ACNS (BACNS) and atypical PACNS. However, BACNS, also named reversible cerebral vasoconstriction syndrome (RCVS), is now recognized as a vasospastic disorder rather than vasculitis; thus, it has been excluded from ACNS.^6^,^7^ Due to the heterogeneity of the atypical PACNS, this term was subsequently replaced by 4 additional subtypes of PACNS based on clinicopathological and angiographical features. Those are lymphocytic PACNS, angiographically defined PACNS, mass-lesion presentation and amyloid-β-related cerebral angiitis.^8^

Practically, in some PACNS cohort studies, the patients were divided by angiograms into 2 groups: large/proximal arteries involved and small/distal arteries involved, since the investigators presumed that the size of the involved vessels was responsible for the clinical differences.^9^,^10^ All angiography-negative cases were defined as small-vessel vasculitis, and angiography-positive cases were defined as middle-vessel vasculitis.^11^ The different classifications of adult PACNS led to the variable clinical appearance, outcome of treatment and prognosis.

## Childhood PACNS

Childhood PACNS (cPACNS) refers to a diagnosis at an age under 18 years old. Three subtypes of cPACNS are proposed, among which two belong to large-medium vessel diseases (angiography-positive), that are non-progressive and progressive large-medium vessel cPACNS, and the third one is small-vessel cPACNS (angiography-negative, biopsy positive). Although cPACNS is infrequent, it is an indispensable cause of childhood vascular strokes.

## Secondary ACNS

The SACNS is a central nervous system vasculitis caused by known etiologies. Systemic vasculitides and vasculitis associated with some systemic diseases, especially immune abnormalities such as lupus vasculitis, rheumatoid vasculitis, Gougerot Sjögren’s syndrome and idiopathic hypereosinophilic syndrome,^4^,^12^ may initiate ACNS. Cancer, certain drugs and infections are also considered to be potential etiologies.

## Pathogenesis of PACNS

Currently, the etiology and pathogenesis of PACNS remain elusive, yet infection and immune disorders are deemed to be the main culprits.

Many infectious agents have been associated with PACNS. Varicella zoster virus (VZV), which was often mentioned in the cases of granulomatous arteritis, was thought to be one of the most important candidates. Other infectious agents, such as Mycoplasma gallisepticum and human immunodeficiency virus (HIV), were also identified in PACNS cases. However, the detailed mechanisms of infection mediating PACNS have not been clarified, and infections are considered more likely to be mimics of PACNS.^13^,^14^

In addition to infection, immunological mechanisms are suggested to play an essential role in PACNS. Langford summarized 3 potential mechanisms that might contribute to the pathogenesis of primary vasculitis syndromes: formation of immune complexes, production of anti-neutrophil cytoplasmic antibodies (ANCA) and pathogenic T-lymphocytes responses with granuloma formation.^15^ However, the evidence of the above-mentioned mechanisms is insufficient in PACNS. The finding of predominant CD45R0+ T lymphocyte infiltration in the biopsy sample of a PACNS patient suggested that T lymphocytes and a potential antigen-specific immune response may be involved in the pathogenesis of PACNS. However, this is the only report that delineated an immunological phenotype of inflammatory aggregators.

**Figure 1:**
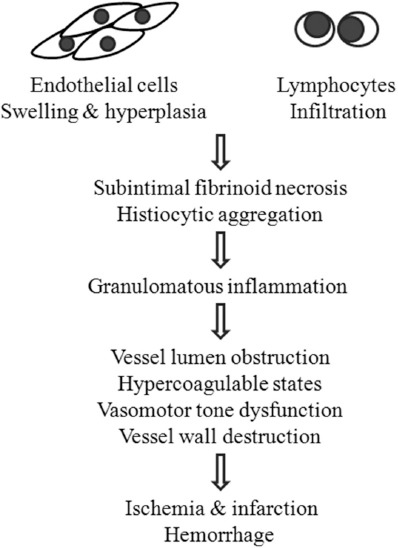
Pathogenesis of PACNS From the point of pathology, the natural processes of PACNS were suggested to follow 3 steps: 1) endothelial cell swelling and hyperplasia, and lymphocytic infiltration; 2) subintimal fibrinoid necrosis of small vessels and histiocytic infiltration; and 3) marked granulomatous inflammation. From the point of pathophysiology, ischemia and infarction or hemorrhage were considered to be results of the inflammation process, which leads to the obstruction of the vessel lumen, hypercoagulable states, vasomotor tone dysfunction, and destruction of the vascular wall.

There are three fundamental histopathological patterns of PACNS in adults, including granulomatous, lymphocytic and necrotizing lesion, which do not typically coexist in one case. A rational explanation is that the natural processes of PACNS begin with the endothelial cells swelling, hyperplasia and lymphocytic infiltration. Then, they develop to subintimal fibrinoid necrosis of small vessels with histiocytic aggregation and finally result in marked granulomatous inflammation (**Figure 1**).^16^ However, the detailed association between the pathology and pathogenesis of PACNS still needs further investigation.

From the point of pathophysiology, neurologic manifestations of ACNS principally result from ischemia and infarction or intracranial hemorrhage. Ischemia is predominantly attributed to the inflammation process, which leads to the obstruction of the vessel lumen, hypercoagulable states and vasomotor tone dysfunction. Moreover, intracranial hemorrhage may also be caused by destruction of the vascular wall.^16^

## Clinical manifestation of ACNS. Epidemic features

The morbidity of PACNS is estimated to be 2.4 cases per one million people per year. Primary ACNS can develop at any age, and the mean age at diagnosis is approximately 50 years.^9^ A variable sexual shift is reported in different studies. Increased mortality has been related to cerebral infarctions and larger/proximal vessel involvement, while better prognosis is associated with the lymphocytic pattern, MRI findings of gadolinium-enhanced cerebral or meninges lesions. With regard to relapses/recurrences, there are no differences between small/distal vessel and large/proximal vessel lesions, which was proven by biopsy or angiography. However, gadolinium-enhanced meninges on an MRI have higher relapse rates.^17^,^18^ Most studies focus on the adult population; however, PACNS affects children as well. There is no specific age distribution in children, but a boy predominance in angiography-positive large-medium vessel cPACNS and girl dominance in angiography-negative small vessel cPACNS were reported.^19^

The epidemiology of SACNS depends on the underlying primary disease. For instance, the brain was rarely affected (11%) in microscopic polyangiitis (MPA) patients, and ACNS did not contribute to its mortality. In the case of granulomatosis with polyangiitis, cerebral small-vessel vasculitis occurred in approximately 4% of patients but often accounted for encephalopathies, seizures and pituitary abnormalities. It is likely that rheumatoid vasculitis and lupus vasculitis seldom trigger ACNS.

## Clinical manifestation

The patients with PACNS show variable manifestations (**Table 1**) due to focal, multifocal or diffuse ischemia or an infarction lesion affecting varied regions of the CNS, which could occur acutely, sub-acutely, recurrently or chronically.

**Table 1:** Common clinical manifestations of PACNS.

Adult PACNS*
Headache
Cognitive dysfunction
Hemiparesis
Consistent neurologic deficit or stroke
Visual symptoms
Transient ischemic attack
Aphasia
Seizures
Ataxia
Intracranial hemorrhage
***Large-medium vessel cPACNS^†^***
Focal neurologic deficits
1. Acute hemiparesis
2. Hemisensory loss
3. Fine motor skill loss
4. Hemifacial weakness
Headaches
Seizures
Diffuse neurologic deficit
***Small-vessel cPACNS***
Any neurologic or psychiatric symptoms

* PACNS - primary angiitis of the central nervous system,

† cPACNS - children primary angiitis of the central nervous system

In the most recent and largest retrospective PACNS study of 163 patients at the Mayo Clinic, the most common symptoms at diagnosis included headache and cognitive dysfunction, which presented in more than half of the patients, followed by hemiparesis 40.5%, persistent neurologic deficit or stroke 40.5% and visual symptoms occurring in 37.4% of the patients. Moreover, headache, cognitive abnormalities and continuous neurologic disorders were the most common initial symptoms, altogether affecting 71% of patients. In addition, other symptoms, such as transient ischemic attack, aphasia, seizures, ataxia, intracranial hemorrhage and systemic symptoms, were reported.^9^ However, de Boysson et al^17^ found a notably higher frequency (83%) of focal neurologic deficit, followed by new-onset headache (54%), cognitive impairment (35%), speech disorders (35%), seizures (33%) and other symptoms. Despite this disparity, a consensus was achieved that patients with a positive biopsy seemed to mainly manifest cognitive dysfunction, whereas angiogram-confirmed PACNS patients have a higher incidence of hemiparesis or persistent neurologic deficit or stroke. Psychiatric symptoms were not identified in most investigations, but de Boysson^17^ reported 13 out of 52 PACNS patients with psychiatric disorders.The clinical manifestations of cPACNS are similar to those of adults. According to the largest published cohort study of cPACNS confirmed by angiography, 62 children with large-medium vessel cPACNS typically presented with focal neurologic deficits including acute hemiparesis (81%), hemisensory loss (79%), fine motor skill loss (73%) and hemifacial weakness (58%). There was no significant difference between non-progressive and progressive cPACNS, excluding hemiparesis. However, headaches, seizures and diffuse neurologic deficit, such as concentration difficulties, cognitive dysfunction, and mood/personality changes, were notably more frequently present in children with progressive cPACNS compared with non-progressive cPACNS. In small-vessel cPACNS, no large cohorts have been published. The frequencies of clinical features from different studies were summarized by Twilt and Benseler:^11^,^20^ headache (50-100%), focal deficits (37–75%), diffuse deficits (17–100%) and seizures (0-100%). Seizures were considered more frequent in children.^16^ However, due to the scarcity of the cases, the frequencies of the above-mentioned manifestations have a wide variation and are not well suited for comparison.

Systemic symptoms are uncommon in both cPACNS and adult PACNS. In contrast, patients with SACNS often suffer from fever, fatigue, myalgia, and arthralgia. In SACNS, the brain involvement is usually a late and rare manifestation of the disease and is always accompanied with other symptoms and signs of the primary disease. Recently, Vera-Lastra et al^21^ conducted a study and compared various aspects, including clinical features, in PACNS vs SACNS. According to their observations, headache was the most frequent neurological manifestation in both groups with similar frequencies; however, the symptom was more severe in PACNS than SACNS patients. The frequencies of neuropathy, cognitive deficit, convulsive crisis, and cerebellar syndrome were also similar in both groups, while more cranial nerve involvement and focal motor deficit were found in PACNS.

## Examination of ACNS. Imaging

Neuroimaging plays an important role in ACNS diagnosis. A variety of imaging diagnostic tests are carried out, including computed tomography (CT), magnetic resonance imaging (MRI), magnetic resonance angiography (MRA), CT angiography, digital subtraction cerebral angiography, ultrasound and nuclear medicine imaging techniques.

## Magnetic resonance imaging

A CT scan is the first choice of radiographic examination for patients with newly onset neurological abnormalities. Nevertheless, MRI is much more sensitive than CT in detecting changes in cerebral vasculitis, except for cerebral hemorrhage. The sensitivity is estimated at more than 75%, even close to 100%,^4^,^21^ while its specificity is poor.

The MRI findings of adults with PACNS are varied and often present as bilateral hyperintense lesions in the cortical and subcortical white matter, deep white matter or gray matter, suggesting infarction or hemorrhage.^22^ A leptomeningeal enhancement and hyperintense T2WI/FLAIR lesion also can be detected.^4^ In the most recent cohort study, Salvarani et al^9^ reported that infarctions were found in 54% of adult PACNS patients, which tended to be multiple, bilateral lesions involving both cortex and subcortex, while intracranial hemorrhage was rarely observed (8%). Elbers et al^23^ found that 91% of patients had bilateral involvement.

In the case of SACNS, hyperintense T2WI/FLAIR lesions in the white matter are also the principal findings, as with PACNS.^21^ Since the findings are non-specific, the interpretation should be performed carefully.

A high-resolution contrast-enhanced vessel wall MRI may help define vessel wall properties and distinguish ACNS from RCVS, the main mimicker of PACNS. In addition, other advanced MRI techniques, including perfusion, spectroscopy, and diffusion weighted imaging (DWI), are also favored in the diagnosis of ACNS.^24^

## Magnetic resonance angiography

For the inadequate resolution of MRA in the detection of small vessel lesions, which are usually present in PACNS, MRA studies were generally thought to provide minimal guidance in the assessment of PACNS,^25^ except for the application in large-vessel cPACNS.^26^ However, a recent study demonstrated a high prevalence (76.8%) of abnormal findings using intracranial time of flight-MRA, suggesting a benefit of this new MRA technique.^22^

## Cerebral angiography

Cerebral angiography has been of great value in the diagnosis and evaluation of ACNS for decades. However, it has inherent risks, with the major risk being embolic stroke.^27^ Typical angiographic findings of cerebral vasculitis include single or multiple focus/foci of stenosis and dilatation, which is known as beading,^28^ while microaneurysms are rarely observed. A recent study from a Mayo Clinic cohort showed that the angiography was positive in 88% of adult PACNS patients, with a predominant presence (95.6%) of multiple bilateral vessel abnormalities, and both large and small vessel were affected: small vessel involvements occurred in 91.2% of the patients, compared with 66.4% for large vessels.^9^ In the cases of angiography-positive cPACNS, the main characteristics of the angiography findings were similar to those of an MRI: unilateral, proximal and within anterior circulation.

Cerebral angiography has a relatively low sensitivity rate given its limited resolution, where involved vessels smaller than 0.2 mm may not be detected. Moreover, the false-positive rate of cerebral angiography was significant (30% to 40%).^24^ Therefore, it is important to combine angiographic results with clinical and laboratory findings to make a reasonable interpretation.

## Laboratory studies. Hematological studies

Hematological studies are unspecific for PACNS. In adult PACNS, hemoglobin levels, white blood counts, platelets counts, acute phase reactants, such as erythrocyte sedimentation rate (ESR), specific serologic tests for systematic vasculitides and infections, such as ANCAs, antinuclear antibodies (ANAs), rheumatoid factor, lupus anticoagulant, serum C3 and C4 and HIV are often negative or within reference.^9^,^29^

Similar results are found in children with angiography-positive cPACNS; however, minor disaccord can sometimes be observed. Notably, the ESR and C-reactive protein levels are likely to increase in children with angiography-negative small vessel vasculitis.^30^ On the other hand, the appearance of hematological abnormalities, such as low leukocytes and platelets, elevated ESR, positive ANAs, anti-dsDNA, ANCAs, low complement, and increased rheumatoid factors, often suggest SACNS or other disease entities. Tuberculosis and VZV can cause granulomatous angiitis, thus, it is proposed that specific evaluations for pathogens should be performed.

## Cerebrospinal fluid analysis (CSF)

Cerebrospinal fluid analysis is non-specific, but critical, in the diagnosis of ACNS and should be performed first, unless it is contraindicated. CSF shows abnormalities in 80-90% of PACNS patients, including a mild lymphocyte-predominant pleocytosis and elevated protein concentration, while the CSF glucose level is normal.^9^ Similar findings are observed in SACNS, but lower than those in PACNS.^21^

Cerebrospinal fluid analysis is very helpful in distinguishing PACNS from RCVS or other angiographic mimics of PACNS, since CSF analysis in patients with RCVS shows mostly normal.^25^ In addition, CSF stains and cultures, a test for anti-VZV IgG antibody and an interferon gamma release assay for Mycobacterium tuberculosis could be applied to exclude infections or malignant diseases.

## Brain-biopsy

A brain biopsy remains the gold standard analysis in the work-up of PACNS, especially in excluding ACNS mimics, particularly infection or malignancy.^31^ It is often performed after a series of less invasive assessments that failed to identify the diagnosis. A brain biopsy may be falsely negative when an unaffected region is sampled due to the focal and segmental distribution of ACNS. Consequently, a single negative biopsy finding does not exclude the diagnosis of ACNS. To decrease the false-negative rate, some strategies can be added, such as targeting of a suspected lesion area with an imaging abnormality and inclusion of leptomeningeal sampling.

Three major patterns of ACNS are observed in a biopsy: granulomatous, necrotizing, and lymphocytic. Mixed types may occur. The histologic patterns are found stable over time suggesting that these patterns do not represent different phases of the disease. This finding contradicts the previously mentioned viewpoint, which was based on serial sections of typical lesions in the spinal cord and proposed that different features of the lesions represented different stages of the disease. Granulomatous vasculitis, the most popular phenotype, is characterized by vasculocentric mononuclear infiltration accompanied with well-formed granulomas and/or multinucleated giant cells through the vessel wall. A β-A4 amyloid deposition is frequently seen in granulomatous vasculitis, but is not unique to this group.^16^ The incidence of lymphocytic vasculitis ranks second and is characterized by marked lymphocytic inflammation with occasional plasma cells typically ranging in multiple layers. Necrotizing vasculitis, which is the least frequent, manifests as transmural fibrinoid necrosis, which resembles the finding in polyarteritis nodosa.^16^ However, in children with small-vessel cPACNS, brain biopsies mostly show intramural lymphocyte (100%) and macrophage (85%) infiltration, surrounding gliosis (100%) and reactive endothelial cells (100%), instead of granulomatous inflammation and multinucleated giant cells.^23^

Appropriate staining and further molecular evaluation should be carried out on the biopsies, especially when lymphocytic infiltration is found. Positive findings may help support a diagnosis of ACNS secondary to infections (e.g., VZV) or malignant causes.

## Diagnosis and differential diagnosis. Diagnostic criteria

There is no published consensus on diagnostic criteria for PACNS; however, 3 essential elements are included: (1) manifestation of CNS angiitis, (2) exclusion of other disorders, and (3) confined to the CNS. The most widely used criteria were proposed by Calabrese and Mallek in 1988.

Since RCVS patients are often mistaken for PACNS as a result of the non-specificity of the CNS angiography, a definite diagnosis should be established only when it is further supported by biopsy, otherwise it is a tentative diagnosis. Recently, Powers^32^ discussed the dispute on whether histologic proofs of angiitis are required and suggested that PACNS should not be entitled without histologic evidence. In addition, modified diagnostic criteria were proposed: (1) histologic demonstration, autopsy criteria for definite diagnosis and biopsy criteria with less certainty, (2) evidence of restriction to the CNS, and (3) ruling out other conditions.

For SACNS, the brain involvement usually occurs in the late stage of the primary disease. Biopsy alone could not clearly differentiate SACNS from PACNS. The diagnosis of SACNS is generally based on the radiological and/or histopathological evidence of ACNS in the setting of a systemic inflammatory or infectious disease or other conditions.^3^,^21^

## Differential diagnosis of PACNS

The differential diagnosis of PACNS are complicated, including SACNS, non-vasculitic autoimmune and inflammatory brain diseases, non-inflammatory vasculopathies and other miscellaneous diseases (**Table 2**).^28^ For suspected PACNS, clinicians should be aware of these mimics to avoid a misdiagnosis.

**Table 2:** Differential diagnosis of PACNS.

***Secondary central nervous system vasculitis***
Systemic vasculitides: Takayasu arteritis, giant cell arteritis, polyarteritis nodosa, Kawasaki disease, granulomatosis with polyangiitis, eosinophilic granulomatosis with polyangiitis, Behçet disease, etc.
Vasculitis associated with systemic diseases: lupus vasculitis, rheumatoid vasculitis, Gougerot Sjögren’s syndrome, etc.
Infections: viral (e.g., herpes zoster, HIV^1^), bacterial (e.g., tuberculosis, syphilis), fungal (e.g., aspergillosis, cryptococcus), mycoplasmal, etc
Cancer-associated: Hodgkin and non-Hodgkin lymphoma, leukemia, etc.
***Nonvasculitic autoimmune and inflammatory brain diseases***
Neuromyelitis optica, N-Methyl-D-aspartate receptor–mediated encephalitis, Susac syndrome, optic neuritis, multiple sclerosis, acute demyelinating encephalomyelitis, Rasmussen encephalitis.
***Non-inflammatory vasculopathies***
RCVS:^2^ Call-Fleming syndrome, postpartum angiopathy, migrainous vasospasm, drug-induced arteritis, BACNS^3^.
Others such as fibromuscular dysplasia, Moyamoya disease, intracranial dissection, radiation vasculopathy, etc.
***Miscellaneous***
thromboembolic disease, bacterial endocarditis, anti-phospholipid syndrome and other hypercoagulable states, cardiac myxoma embolism, cholesterol atheroembolism, hemoglobin disorders, etc.

HIV - human immunodeficiency virus, RCVS - reversible cerebral vasoconstriction syndrome, BACNS - benign angiitis of the central nervous system

## Therapeutic strategies for ACNS. Treatment of PACNS

There are no well-randomized clinical trials to define the optimal management of PACNS and its subtypes, and regimens for PACNS are majorly derived from strategies proposed in other vasculitis.

## Treatment for PACNS in adults

It is suggested that corticosteroid with or without cyclophosphamide may result in a favorable response in adults.^18^,^25^ Based on the data and the experience, Salvarani et al^9^ proposed a treatment algorithm for adult PACNS. For patients with small/distal vessel PACNS, an initial oral prednisone (1 mg/kg per day) is recommended. Methylprednisolone bolus therapy (1000 mg per day for 3-5 days) should be considered in the cases of acute onset. If the response to the treatment is beneficial, prednisone can be weaned gradually; otherwise, cyclophosphamide should be added. Other immunosuppressive agents, such as azathioprine and mycophenolate mofetil, have been shown to be effective and thus, can replace the more toxic cyclophosphamide for induction as well as for maintenance therapy (azathioprine 1-2 mg/kg per day; mycophenolate mofetil 1-2 g per day) combined with low-dose prednisone. Maintenance therapy was also recommended by de Boysson et al^33^ since it was suggested to be correlated with better outcomes and fewer relapses.

In patients diagnosed with large/proximal vessel PACNS, who typically undergo a rapidly progressive clinical course and respond poorly to therapy, a combination of methylprednisolone bolus therapy, oral prednisone and cyclophosphamide (oral 2 mg/kg daily for 3-6 months, or intravenous 0.75 g/m^2^ monthly for 6 months) is recommended to induce remission. Maintenance therapy is the same as small/distal vessel PACNS.

During the administration of a combination of cyclophosphamide and glucocorticoid, prophylaxis for opportunistic infections, especially targeting Pneumocystis jirovecii, is recommended in immunosuppressed patients. Furthermore, supplements of bisphosphonates, calcium and vitamin D are suggested to prevent osteoporosis and fractures.^25^ In addition, biologic agents, including tumor necrosis factor-α blockers and rituximab, may be options for refractory patients.

## Treatment for childhood PACNS

A treatment protocol for children diagnosed with small vessel cPACNS is recommended to control neurological manifestations and to improve neurological outcome.^30^ The induction therapy lasts for 6 months, comprising 7 pulses of intravenous cyclophosphamide (500–750 mg/m^2^, every 4 weeks) with co-trimoxazole prophylaxis and oral prednisone daily (initially starting 2 mg/kg/day and weaned every 4 weeks). The maintenance therapy usually lasts 18 months and consists of mycophenolate mofetil (800–1200 mg/m² per day), which is preferred, or azathioprine (2–3 mg/kg per day) and prednisone weaned every 4 weeks. Anticonvulsants and antipsychotics are added if necessary.

As for non-progressive angiography-positive large-vessel cPACNS, a 5-day intravenous methylprednisolone treatment followed by 3-month tapering dose of oral glucocorticoids is suggested. Large-vessel cPACNS rarely relapses during dose tapering.^34^ Anticoagulation and/or antiplatelet therapy needs to be employed simultaneously.^19^

In progressive cPACNS, induction therapy with high-dose corticosteroids and cyclophosphamide (6 months) following mycophenolate mofetil maintenance (18 months) and antithrombotic therapy is recommended.^19^ Co-trimoxazole, calcium and vitamin D are beneficial for prophylaxis against infection and osteoporosis in children during treatment.

## Treatment of SACNS

The treatment of SACNS should be based on the primary disease. In most cases of ACNS secondary to systemic vasculitides, the recommended therapies are the same as PACNS, including intravenous cyclophosphamide plus corticosteroid.^21^,^35^ Biological agents have also been shown to be beneficial in difficult-to-treat patients with SACNS.^36^ While in the cases of infection-associated vasculitis, antimicrobial or antiretroviral therapy should be first considered,^37^ and adjunctive immunosuppressive therapy must be done with caution.

In conclusion, the etiologies and pathogenesis of PACNS remain obscure. Because ACNS has variable clinical presentations, diagnosis is mostly established from neuroimaging and brain-biopsy. The treatment with corticosteroid with or without cyclophosphamide is suggested; however, the optimal strategy requires further confirmation.
